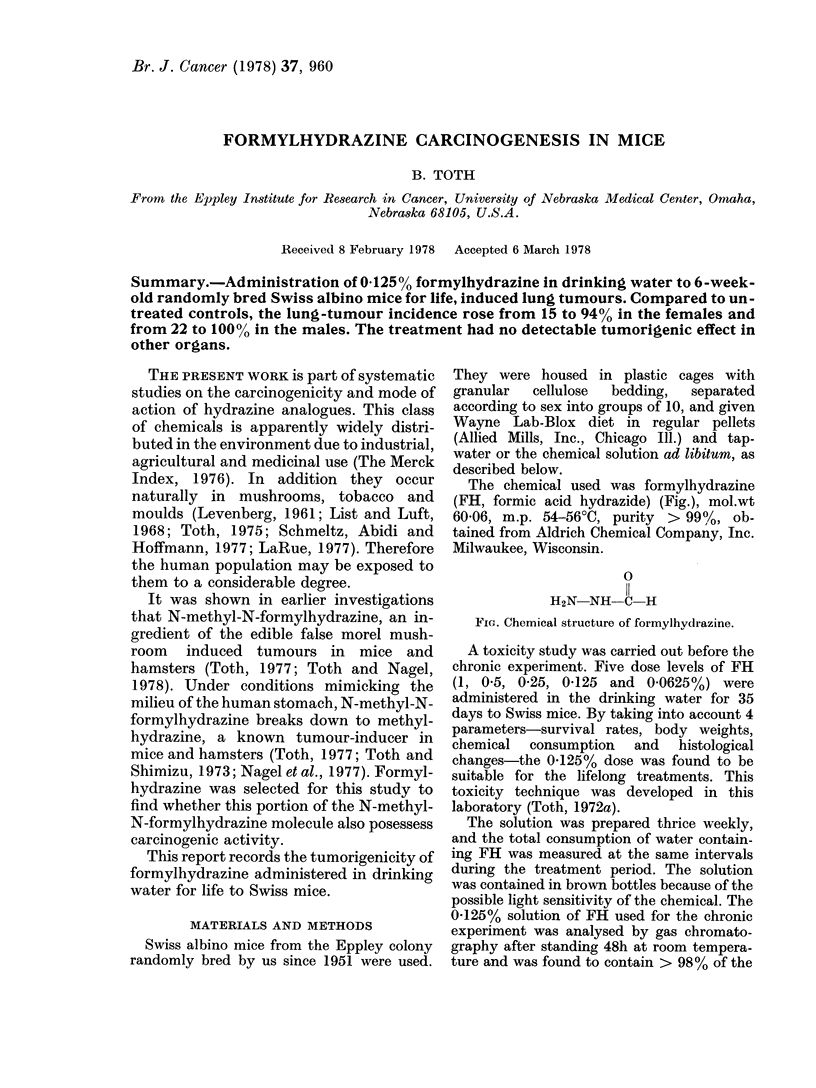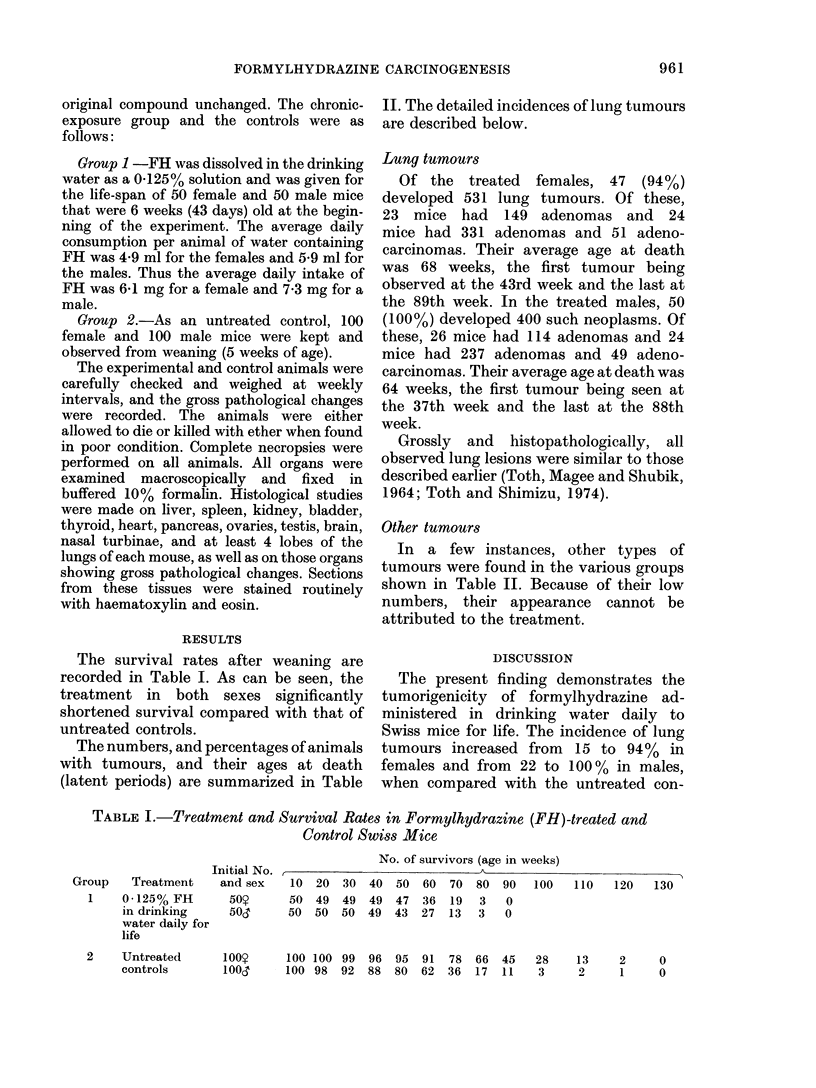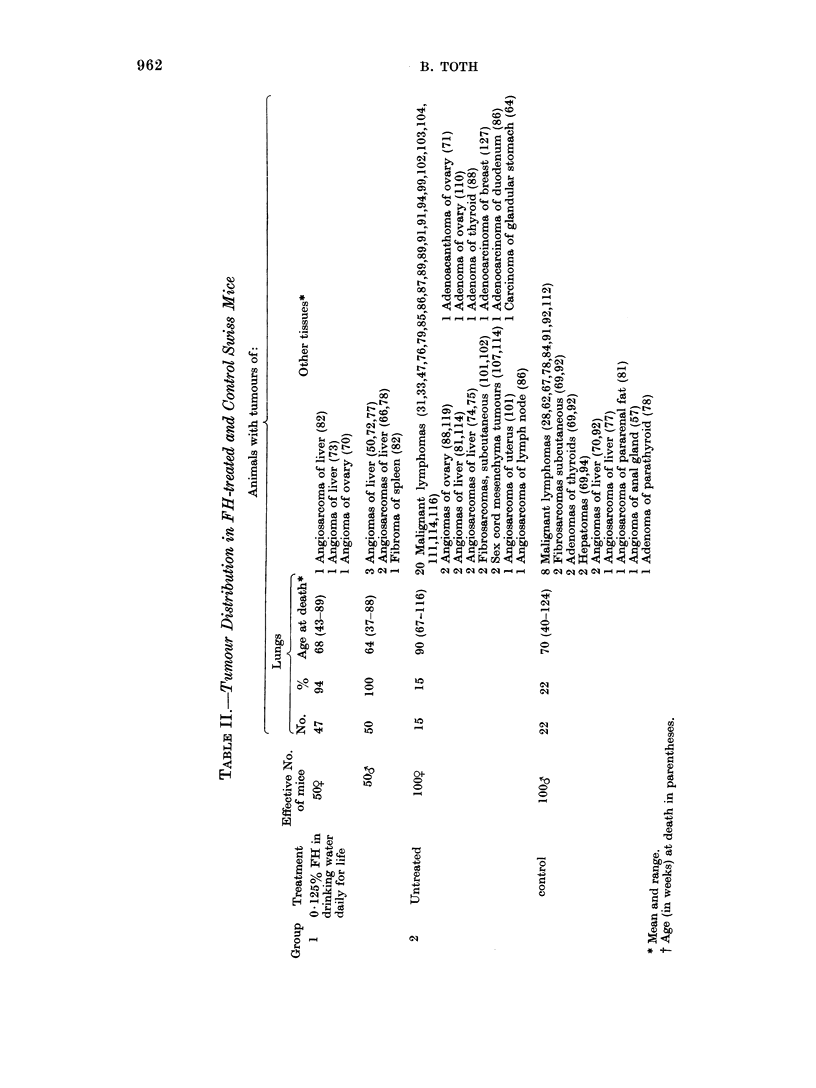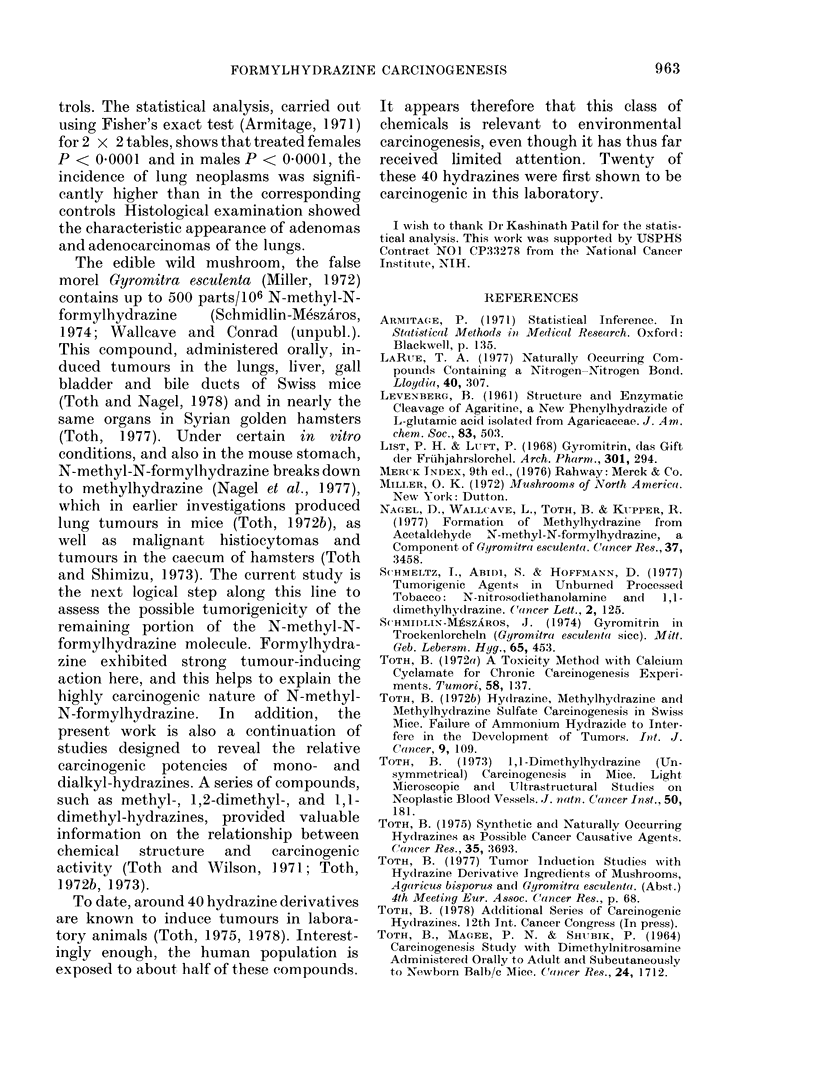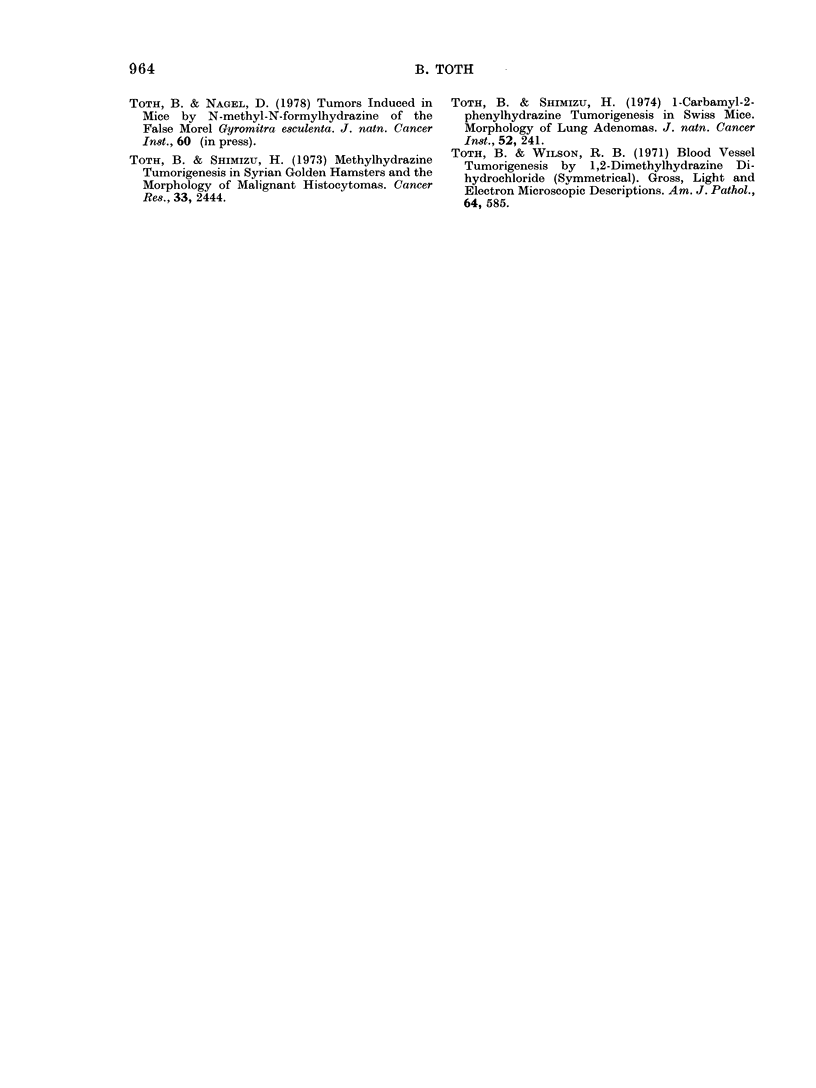# Formylhydrazine carcinogenesis in mice.

**DOI:** 10.1038/bjc.1978.140

**Published:** 1978-06

**Authors:** B. Toth

## Abstract

Administration of 0.125% formylhydrazine in drinking water to 6-week-old randomly bred Swiss albino mice for life, induced lung tumours. Compared to untreated controls, the lung-tumour incidence rose from 15 to 94% in the females and from 22 to 100% in the males. The treatment had no detectable tumorigenic effect in other organs.


*Br. J. Cancer* (1978) **37**, 960

### FORMYLHYDRAZINE CARCINOGENESIS IN MICE

B. TOTH

*From the Eppley Institute for Research in Cancer, University of Nebraska Medical Center, Omaha, Nebraska 68105, U.S.A.*

Received 8 February 1978 Accepted 6 March 1978

**Summary.—Administration of 0·125% formylhydrazine in drinking water to 6-week-old randomly bred Swiss albino mice for life, induced lung tumours. Compared to untreated controls, the lung-tumour incidence rose from 15 to 94% in the females and from 22 to 100% in the males. The treatment had no detectable tumorigenic effect in other organs.**

THE PRESENT WORK is part of systematic studies on the carcinogenicity and mode of action of hydrazine analogues. This class of chemicals is apparently widely distributed in the environment due to industrial, agricultural and medicinal use (The Merck Index, 1976). In addition they occur naturally in mushrooms, tobacco and moulds (Levenberg, 1961; List and Luft, 1968; Toth, 1975; Schmeltz, Abidi and Hoffmann, 1977; LaRue, 1977). Therefore the human population may be exposed to them to a considerable degree.

It was shown in earlier investigations that N-methyl-N-formylhydrazine, an ingredient of the edible false morel mushroom induced tumours in mice and hamsters (Toth, 1977; Toth and Nagel, 1978). Under conditions mimicking the milieu of the human stomach, N-methyl-N-formylhydrazine breaks down to methylhydrazine, a known tumour-inducer in mice and hamsters (Toth, 1977; Toth and Shimizu, 1973; Nagel *et al.*, 1977). Formylhydrazine was selected for this study to find whether this portion of the N-methyl-N-formylhydrazine molecule also posesesss carcinogenic activity.

This report records the tumorigenicity of formylhydrazine administered in drinking water for life to Swiss mice.

### MATERIALS AND METHODS

Swiss albino mice from the Eppley colony randomly bred by us since 1951 were used. They were housed in plastic cages with granular cellulose bedding, separated according to sex into groups of 10, and given Wayne Lab-Blox diet in regular pellets (Allied Mills, Inc., Chicago Ill.) and tap-water or the chemical solution *ad libitum*, as described below.

The chemical used was formylhydrazine (FH, formic acid hydrazide) (Fig.), mol.wt 60·06, m.p. 54–56°C, purity > 99%, obtained from Aldrich Chemical Company, Inc. Milwaukee, Wisconsin.

FIG. Chemical structure of formylhydrazine.

A toxicity study was carried out before the chronic experiment. Five dose levels of FH (1, 0·5, 0·25, 0·125 and 0·0625%) were administered in the drinking water for 35 days to Swiss mice. By taking into account 4 parameters—survival rates, body weights, chemical consumption and histological changes—the 0·125% dose was found to be suitable for the lifelong treatments. This toxicity technique was developed in this laboratory (Toth, 1972*a*).

The solution was prepared thrice weekly, and the total consumption of water containing FH was measured at the same intervals during the treatment period. The solution was contained in brown bottles because of the possible light sensitivity of the chemical. The 0·125% solution of FH used for the chronic experiment was analysed by gas chromatography after standing 48h at room temperature and was found to contain > 98% of the original compound unchanged. The chronic-exposure group and the controls were as follows:

*Group 1* —FH was dissolved in the drinking water as a 0·125% solution and was given for the life-span of 50 female and 50 male mice that were 6 weeks (43 days) old at the beginning of the experiment. The average daily consumption per animal of water containing FH was 4·9 ml for the females and 5·9 ml for the males. Thus the average daily intake of FH was 6·1 mg for a female and 7·3 mg for a male.

*Group 2.*—As an untreated control, 100 female and 100 male mice were kept and observed from weaning (5 weeks of age).

The experimental and control animals were carefully checked and weighed at weekly intervals, and the gross pathological changes were recorded. The animals were either allowed to die or killed with ether when found in poor condition. Complete necropsies were performed on all animals. All organs were examined macroscopically and fixed in buffered 10% formalin. Histological studies were made on liver, spleen, kidney, bladder, thyroid, heart, pancreas, ovaries, testis, brain, nasal turbinae, and at least 4 lobes of the lungs of each mouse, as well as on those organs showing gross pathological changes. Sections from these tissues were stained routinely with haematoxylin and eosin.

### RESULTS

The survival rates after weaning are recorded in Table I. As can be seen, the treatment in both sexes significantly shortened survival compared with that of untreated controls.

The numbers, and percentages of animals with tumours, and their ages at death (latent periods) are summarized in Table II. The detailed incidences of lung tumours are described below.

### *Lung tumours*

Of the treated females, 47 (94%) developed 531 lung tumours. Of these, 23 mice had 149 adenomas and 24 mice had 331 adenomas and 51 adenocarcinomas. Their average age at death was 68 weeks, the first tumour being observed at the 43rd week and the last at the 89th week. In the treated males, 50 (100%) developed 400 such neoplasms. Of these, 26 mice had 114 adenomas and 24 mice had 237 adenomas and 49 adenocarcinomas. Their average age at death was 64 weeks, the first tumour being seen at the 37th week and the last at the 88th week.

Grossly and histopathologically, all observed lung lesions were similar to those described earlier (Toth, Magee and Shubik, 1964; Toth and Shimizu, 1974).

### *Other tumours*

In a few instances, other types of tumours were found in the various groups shown in Table II. Because of their low numbers, their appearance cannot be attributed to the treatment.

### DISCUSSION

The present finding demonstrates the tumorigenicity of formylhydrazine administered in drinking water daily to Swiss mice for life. The incidence of lung tumours increased from 15 to 94% in females and from 22 to 100% in males, when compared with the untreated con-

TABLE I.—*Treatment and Survival Rates in Formylhydrazine (FH)-treated and Control Swiss Mice*

| Group | Treatment | Initial No. and sex | No. of survivors (age in weeks) 10 | 20 | 30 | 40 | 50 | 60 | 70 | 80 | 90 | 100 | 110 | 120 | 130 |
|---|---|---|---|---|---|---|---|---|---|---|---|---|---|---|---|
| 1 | 0·125% FH in drinking water daily for life | 50♀ | 50 | 49 | 49 | 49 | 47 | 36 | 19 | 3 | 0 | | | | |
| | | 50♂ | 50 | 50 | 50 | 49 | 43 | 27 | 13 | 3 | 0 | | | | |
| 2 | Untreated controls | 100♀ | 100 | 100 | 99 | 96 | 95 | 91 | 78 | 66 | 45 | 28 | 13 | 2 | 0 |
| | | 100♂ | 100 | 98 | 92 | 88 | 80 | 62 | 36 | 17 | 11 | 3 | 2 | 1 | 0 |

TABLE II.—*Tumour Distribution in FH-treated and Control Swiss Mice*

| Group | Treatment | Effective No. of mice | Animals with tumours of: Lungs No. | % | Age at death* | Other tissues* |
|---|---|---|---|---|---|---|
| 1 | 0·125% FH in drinking water daily for life | 50♀ | 47 | 94 | 68 (43–89) | 1 Angiosarcoma of liver (82)<br>1 Angioma of liver (73)<br>1 Angioma of ovary (70) |
| | | 50♂ | 50 | 100 | 64 (37–88) | 3 Angiomas of liver (50,72,77)<br>2 Angiosarcomas of liver (66,78)<br>1 Fibroma of spleen (82) |
| 2 | Untreated | 100♀ | 15 | 15 | 90 (67–116) | 20 Malignant lymphomas (31,33,47,76,79,85,86,87,89,89,91,91,94,99,102,103,104, 111,114,116)<br>2 Angiomas of ovary (88,119)<br>2 Angiomas of liver (81,114)<br>2 Angiosarcomas of liver (74,75)<br>2 Fibrosarcomas, subcutaneous (101,102)<br>2 Sex cord mesenchyma tumours (107,114)<br>1 Angiosarcoma of uterus (101)<br>1 Angiosarcoma of lymph node (86)<br>1 Adenoacanthoma of ovary (71)<br>1 Adenoma of ovary (110)<br>1 Adenoma of thyroid (88)<br>1 Adenocarcinoma of breast (127)<br>1 Adenocarcinoma of duodenum (86)<br>1 Carcinoma of glandular stomach (64) |
| | control | 100♂ | 22 | 22 | 70 (40–124) | 8 Malignant lymphomas (28,62,67,78,84,91,92,112)<br>2 Fibrosarcomas subcutaneous (69,92)<br>2 Adenomas of thyroids (69,92)<br>2 Hepatomas (69,94)<br>2 Angiomas of liver (70,92)<br>1 Angiosarcoma of liver (77)<br>1 Angiosarcoma of pararenal fat (81)<br>1 Angioma of anal gland (57)<br>1 Adenoma of parathyroid (78) |

* Mean and range.

† Age (in weeks) at death in parentheses.

trols. The statistical analysis, carried out using Fisher's exact test (Armitage, 1971) for 2 × 2 tables, shows that treated females $P < 0{\cdot}0001$ and in males $P < 0{\cdot}0001$, the incidence of lung neoplasms was significantly higher than in the corresponding controls Histological examination showed the characteristic appearance of adenomas and adenocarcinomas of the lungs.

The edible wild mushroom, the false morel *Gyromitra esculenta* (Miller, 1972) contains up to 500 parts/$10^6$ N-methyl-N-formylhydrazine (Schmidlin-Mészáros, 1974; Wallcave and Conrad (unpubl.). This compound, administered orally, induced tumours in the lungs, liver, gall bladder and bile ducts of Swiss mice (Toth and Nagel, 1978) and in nearly the same organs in Syrian golden hamsters (Toth, 1977). Under certain *in vitro* conditions, and also in the mouse stomach, N-methyl-N-formylhydrazine breaks down to methylhydrazine (Nagel *et al.*, 1977), which in earlier investigations produced lung tumours in mice (Toth, 1972*b*), as well as malignant histiocytomas and tumours in the caecum of hamsters (Toth and Shimizu, 1973). The current study is the next logical step along this line to assess the possible tumorigenicity of the remaining portion of the N-methyl-N-formylhydrazine molecule. Formylhydrazine exhibited strong tumour-inducing action here, and this helps to explain the highly carcinogenic nature of N-methyl-N-formylhydrazine. In addition, the present work is also a continuation of studies designed to reveal the relative carcinogenic potencies of mono- and dialkyl-hydrazines. A series of compounds, such as methyl-, 1,2-dimethyl-, and 1,1-dimethyl-hydrazines, provided valuable information on the relationship between chemical structure and carcinogenic activity (Toth and Wilson, 1971; Toth, 1972*b*, 1973).

To date, around 40 hydrazine derivatives are known to induce tumours in laboratory animals (Toth, 1975, 1978). Interestingly enough, the human population is exposed to about half of these compounds. It appears therefore that this class of chemicals is relevant to environmental carcinogenesis, even though it has thus far received limited attention. Twenty of these 40 hydrazines were first shown to be carcinogenic in this laboratory.

I wish to thank Dr Kashinath Patil for the statistical analysis. This work was supported by USPHS Contract NO1 CP33278 from the National Cancer Institute, NIH.

### REFERENCES

Armitage, P. (1971) Statistical Inference. In *Statistical Methods in Medical Research.* Oxford: Blackwell, p. 135.

LaRue, T. A. (1977) Naturally Occurring Compounds Containing a Nitrogen–Nitrogen Bond. *Lloydia*, **40**, 307.

Levenberg, B. (1961) Structure and Enzymatic Cleavage of Agaritine, a New Phenylhydrazide of L-glutamic acid isolated from Agaricaceae. *J. Am. chem. Soc.*, **83**, 503.

List, P. H. & Luft, P. (1968) Gyromitrin, das Gift der Frühjahrslorchel. *Arch. Pharm.*, **301**, 294.

Merck Index, 9th ed., (1976) Rahway: Merck & Co.

Miller, O. K. (1972) *Mushrooms of North America.* New York: Dutton.

Nagel, D., Wallcave, L., Toth, B. & Kupper, R. (1977) Formation of Methylhydrazine from Acetaldehyde N-methyl-N-formylhydrazine, a Component of *Gyromitra esculenta. Cancer Res.*, **37**, 3458.

Schmeltz, I., Abidi, S. & Hoffmann, D. (1977) Tumorigenic Agents in Unburned Processed Tobacco: N-nitrosodiethanolamine and 1,1-dimethylhydrazine. *Cancer Lett.*, **2**, 125.

Schmidlin-Mészáros, J. (1974) Gyromitrin in Trockenlorcheln (*Gyromitra esculenta* sicc). *Mitt. Geb. Lebersm. Hyg.*, **65**, 453.

Toth, B. (1972*a*) A Toxicity Method with Calcium Cyclamate for Chronic Carcinogenesis Experiments. *Tumori*, **58**, 137.

Toth, B. (1972*b*) Hydrazine, Methylhydrazine and Methylhydrazine Sulfate Carcinogenesis in Swiss Mice. Failure of Ammonium Hydrazide to Interfere in the Development of Tumors. *Int. J. Cancer*, **9**, 109.

Toth, B. (1973) 1,1-Dimethylhydrazine (Unsymmetrical) Carcinogenesis in Mice. Light Microscopic and Ultrastructural Studies on Neoplastic Blood Vessels. *J. natn. Cancer Inst.*, **50**, 181.

Toth, B. (1975) Synthetic and Naturally Occurring Hydrazines as Possible Cancer Causative Agents. *Cancer Res.*, **35**, 3693.

Toth, B. (1977) Tumor Induction Studies with Hydrazine Derivative Ingredients of Mushrooms, *Agaricus bisporus* and *Gyromitra esculenta.* (Abst.) *4th Meeting Eur. Assoc. Cancer Res.*, p. 68.

Toth, B. (1978) Additional Series of Carcinogenic Hydrazines. 12th Int. Cancer Congress (In press).

Toth, B., Magee, P. N. & Shubik, P. (1964) Carcinogenesis Study with Dimethylnitrosamine Administered Orally to Adult and Subcutaneously to Newborn Balb/c Mice. *Cancer Res.*, **24**, 1712.

TOTH, B. & NAGEL, D. (1978) Tumors Induced in Mice by N-methyl-N-formylhydrazine of the False Morel *Gyromitra esculenta*. *J. natn. Cancer Inst.*, **60** (in press).

TOTH, B. & SHIMIZU, H. (1973) Methylhydrazine Tumorigenesis in Syrian Golden Hamsters and the Morphology of Malignant Histocytomas. *Cancer Res.*, **33**, 2444.

TOTH, B. & SHIMIZU, H. (1974) 1-Carbamyl-2-phenylhydrazine Tumorigenesis in Swiss Mice. Morphology of Lung Adenomas. *J. natn. Cancer Inst.*, **52**, 241.

TOTH, B. & WILSON, R. B. (1971) Blood Vessel Tumorigenesis by 1,2-Dimethylhydrazine Dihydrochloride (Symmetrical). Gross, Light and Electron Microscopic Descriptions. *Am. J. Pathol.*, **64**, 585.